# A randomized, controlled trial to evaluate the effect of an anti-interleukin-9 monoclonal antibody in adults with uncontrolled asthma

**DOI:** 10.1186/1465-9921-14-93

**Published:** 2013-09-19

**Authors:** Chad K Oh, Richard Leigh, Kimmie K McLaurin, Keunpyo Kim, Micki Hultquist, Nestor A Molfino

**Affiliations:** 1 MedImmune, One MedImmune Way, Gaithersburg, MD, USA; 2 Department of Medicine, University of Calgary, Calgary, Alberta, Canada; 3 Currently at KaloBios Pharmaceuticals, Inc, South San Francisco, CA, USA

**Keywords:** Asthma, Anti-IL-9 monoclonal antibody, MEDI-528

## Abstract

**Background:**

Preclinical studies suggest that interleukin-9 may be a central mediator in the development and maintenance of airway inflammation in asthma. The aim of this study was therefore to evaluate the effects of MEDI-528, an anti-interleukin-9 monoclonal antibody, in adults with confirmed uncontrolled moderate-to-severe asthma.

**Methods:**

In this prospective double-blind, multicenter, parallel-group study, 329 subjects were randomized (1:1:1:1) to subcutaneous placebo or MEDI-528 (30, 100, 300 mg) every 2 weeks for 24 weeks, in addition to their usual asthma medications. The primary endpoint was change in mean Asthma Control Questionnaire-6 (ACQ-6) score at week 13. Secondary endpoints included weighted asthma exacerbation rates and pre-bronchodilator forced expiratory volume in 1 second (FEV_1_) at weeks 13 and 25, as well as Asthma Quality of Life Questionnaire scores at weeks 12 and 25 and the safety of MEDI-528 throughout the study period. The primary endpoint was analyzed using analysis of covariance.

**Results:**

The study population (n = 327) was predominantly female (69%) with a mean age of 43 years (range 18–65). The mean (SD) baseline ACQ-6 score for placebo (n = 82) and combined MEDI-528 (n = 245) was 2.8 (0.7) and 2.8 (0.8); FEV_1_ % predicted was 70.7% (15.9) and 71.5% (16.7). Mean (SD) change from baseline to week 13 in ACQ-6 scores for placebo vs combined MEDI-528 groups was −1.2 (1.0) vs −1.2 (1.1) (p = 0.86). Asthma exacerbation rates (95% CI) at week 25 for placebo vs MEDI-528 were 0.58 (0.36–0.88) vs 0.49 (0.37–0.64) exacerbations/subject/year (p = 0.52). No significant improvements in FEV_1_ % predicted were observed between the placebo and MEDI-528 groups. Adverse events were comparable for placebo (82.9%) and MEDI-528 groups (30 mg, 76.5%; 100 mg, 81.9%; 300 mg, 85.2%). The most frequent were asthma (placebo vs MEDI-528, 30.5% vs 33.5%), upper respiratory tract infection (14.6% vs 17.1%), and headache (9.8% vs 9.8%).

**Conclusions:**

The addition of MEDI-528 to existing asthma controller medications was not associated with any improvement in ACQ-6 scores, asthma exacerbation rates, or FEV_1_ values, nor was it associated with any major safety concerns.

**Trial registration:**

ClinicalTrials.gov: NCT00968669.

## Background

Asthma is a chronic inflammatory airway disease characterized by variable airflow limitation, airway hyperresponsiveness, mucus hypersecretion, and structural changes to the airways that include proliferation of smooth muscle [1]. Interleukin (IL)-9, a multifunctional cytokine produced by type 2 T helper cells (Th2), lymphocytes, and mast cells, is proposed to be a central mediator in the pathogenesis of asthma. Interest in IL-9 was first triggered by genetic linkage studies [2] and supported by the finding that expression of IL-9 and its associated receptor is higher in the airways of subjects with asthma compared with healthy controls [3,4]. Further evidence has come from *in vitro* studies showing that IL-9 enhances the growth and/or activity of a variety of cell types and pro-inflammatory and pro-fibrotic mediators that are implicated in the pathogenesis of asthma [5]. IL-9 induced the release of Th2-associated chemokines in cultured human airway smooth muscle cells [6] and enhanced the stem cell factor-dependent growth of human mast cell progenitors, particularly those from children with asthma [7]. Another study reported that IL-9 upregulated mucin expression in human lung cells, suggesting that it is involved in the control of mucus secretion [8].

Preclinical studies in animal models of asthma support a contributing role for an IL-9 mast cell axis in the immunopathology of asthma [5]. In one study, anti-IL-9 antibody treatment had a protective effect against airway remodeling in mouse models of airway inflammation, together with a concomitant reduction in the number and activation of mature mast cells [9]. Furthermore, impaired lung function related to airway remodeling was reversed by IL-9 neutralization. Taken together, evidence from these and other experimental studies indicated that targeting IL-9 may offer a novel approach to the treatment of asthma.

MEDI-528 is a humanized immunoglobulin G1 monoclonal antibody that binds to IL-9 [10], and hence reduces the activity of a variety of cell types implicated in asthma pathogenesis [5]. Preliminary clinical studies in healthy adults and subjects with mild or mild-to-moderate asthma have demonstrated that MEDI-528 administered subcutaneously has linear pharmacokinetics and an acceptable safety profile with no reports of serious adverse events (SAEs) [11,12]. One study in subjects with mild asthma showed that MEDI-528 had no effect on pulmonary function or the use of rescue medication, although there were positive trends for improvement in asthma symptom scores and for a reduction in the number of asthma exacerbations [11]. Another study in a small number of subjects with mild-to-moderate asthma suggested that MEDI-528 may decrease sensitivity to exercise-induced bronchoconstriction that is dependent on mast cell degranulation [11]. We now report the results from a larger study (ClinicalTrials.gov, NCT00968669) that was designed to further investigate the potential clinical benefits of MEDI-528 in treating subjects with asthma. The primary objective of this study was to evaluate the effect of multiple dose subcutaneous administration of MEDI-528 on symptom control in adults with uncontrolled, moderate-to-severe, persistent asthma.

## Methods

### Subjects

Eligible subjects were aged 18–65 years with body mass index 18–35 kg/m^2^ and a clinical diagnosis of asthma, confirmed by pre-bronchodilator forced expiratory volume in 1 second (FEV_1_) ≥ 40% predicted and post-bronchodilator FEV_1_ reversibility ≥ 12% and ≥ 200 mL.

Subjects were required to have poor asthma symptom control [1], as demonstrated by an Asthma Control Questionnaire-6 (ACQ-6) score ≥ 1.5 [13], daytime symptoms on ≥ 2 days/week, night-time awakening ≥ 1 night/week, rescue medication use on ≥ 2 days/week, and ≥ 1 asthma exacerbation in the past year. Subjects were either currently taking medium to high-dose inhaled corticosteroids (ICS) or were eligible to take them based on Expert Panel Report 3 guidelines, and were started on medium to high-dose ICS at the start of the run-in phase of the study [1]. Key exclusion criteria were the presence of other lung diseases, severe depression or history of suicidal behavior, tobacco smoking (≥ 10 pack-years), recent illness or infection, or clinically significant electrocardiogram (ECG) abnormalities. Treatment with any other biologic agent or immunosuppressive medication (apart from ≤ 10 mg/day oral prednisone or equivalent, or inhaled or topical corticosteroids) was not allowed during the month prior to and throughout the study. Subjects were permitted to receive a short burst of systemic corticosteroid therapy or be hospitalized to control an asthma exacerbation.

All subjects provided written informed consent before participating in the study. The protocol was approved by an Independent Ethics Committee at each study center, and the study was conducted in accordance with International Conference on Harmonisation Guidance for Good Clinical Practice and the Declaration of Helsinki.

### Study design and randomization

**Figure 1:**
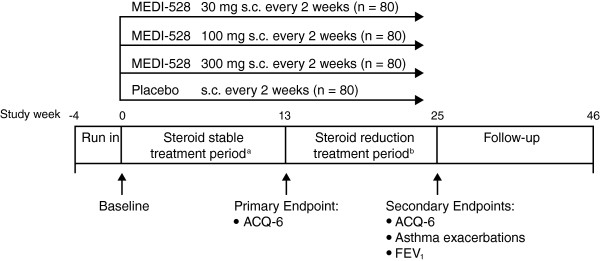
**Study design. **^a^Inhaled corticosteroids at stable dose: fluticasone/salmeterol (500 μg/50 μg or 250 μg/50 μg) or budesonide/formoterol (160 μg/4.5 μg). ^b^Subjects with clinically stable asthma could reduce their inhaled corticosteroid dose to: fluticasone/salmeterol (250 μg/50 μg or 100 μg/50 μg) or budesonide/formoterol (80 μg/4.5 μg). Abbreviations: ACQ-6, Asthma Control Questionnaire-6; FEV_1_, Forced expiratory volume in 1 second; s.c, Subcutaneous.

This phase 2b, randomized, double-blind, placebo-controlled, parallel group study was conducted at 53 sites in North America, Central America, South America, and Asia between October 2009 and November 2011. The study consisted of a 4-week screening period, a 13-week steroid stable treatment period, an 11-week steroid reduction treatment period, and a 22-week follow-up period (Figure 1). The ICS dosage was optimized and stabilized during the screening period, maintained unchanged throughout the steroid-stable treatment period, and was reduced in subjects with clinically stable asthma during the steroid-reduction treatment period. Subjects were seen at the clinic twice during the screening period (weeks −4 and −2), at two-weekly intervals during the treatment period (weeks 0 to 24) with an additional visit at week 13, and then at four-weekly intervals during the post-treatment follow-up period (weeks 25 to 46).

Subjects were randomized (1:1:1:1) using an interactive voice response system to receive placebo or one of three doses of MEDI-528 (30, 100, or 300 mg) subcutaneously every 2 weeks for 24 weeks (13 doses), in addition to their usual asthma medications. Because MEDI-528 and placebo are visually distinct, study medication was administered at the clinic by unblinded personnel who were not otherwise involved in the study in order to maintain blinding.

Randomization was stratified by asthma status (atopic or non-atopic) and ICS dose (medium or high). High-dose ICS was defined as 1 puff twice a day of fluticasone/salmeterol (500 μg/50 μg) and medium dose ICS was defined as 1 puff twice a day fluticasone/sal meterol (250 μg/50 μg) or 2 puffs twice a day budesonide/formoterol (160 μg/4.5 μg). Only subjects who were taking medium- to high-dose ICS at screening were enrolled. Subjects who were uncontrolled while receiving medium-dose ICS were enrolled to the medium-dose or high-dose ICS group at the study physician’s discretion depending on their level of asthma control, and subjects receiving high-dose ICS were enrolled to the high-ICS group. Randomization of subjects with non-atopic asthma was restricted to ensure that a minimum of 160 subjects with atopic asthma were included in the study.

### Primary and secondary outcomes

The primary efficacy outcome was change from baseline in mean ACQ-6 score at week 13 among individual MEDI-528 treatment groups and placebo. Secondary outcomes included change from baseline in mean ACQ-6 score at week 25, asthma exacerbation rates (week 25), pre-bronchodilator FEV_1_ (weeks 13 and 25), health-related quality of life (Asthma Quality of Life Questionnaire (Standardized version) [AQLQ(S)] scores; weeks 12 and 25), and the safety of MEDI-528 throughout the study period.

### Assessments

#### Clinical activity

The ACQ-6 was completed at home on a weekly basis, using an electronic patient-reported outcome device. The occurrence of any asthma exacerbations during the study was recorded by the study physician at each clinic visit. Asthma exacerbation was defined as a progressive increase in asthma symptoms (cough, wheeze, chest tightness, and/or shortness of breath) with a reduction from baseline or best previous measurement of ≥ 20% in FEV_1_ or peak expiratory flow (PEF) rate that did not resolve after the initiation of rescue medication and resulted in a prescription for, or administration of, systemic corticosteroid burst therapy. An exacerbation was considered to be resolved when the subject’s asthma symptoms had diminished, and PEF or FEV_1_ returned to > 80% of baseline for ≥ 7 days after completion of systemic corticosteroid burst therapy. Pre-bronchodilator spirometry was conducted according to the American Thoracic Society/European Respiratory Society guidelines [14]. All spirometry assessments for each subject were performed at the same time of day (± 1 hour) and in the same manner. Between three and eight forced expiratory efforts were made at each session and the maximum FEV_1_ of the two best efforts was recorded.

#### Health-related quality of life

The AQLQ(S) was completed every 4 weeks at home using the electronic patient-reported outcome device.

#### Safety profile

Details of any adverse events (AEs) and SAEs were recorded. AEs were defined as any untoward medical occurrence that occurred after initiation of study medication. Blood and urine samples were collected at screening, prior to the first dose, at 4-weekly intervals during the treatment period, and 8-weekly intervals during the follow-up period for routine clinical laboratory evaluations. Vital signs (blood pressure, heart rate, temperature, and respiratory rate) were monitored at every clinic visit. A computerized 12-lead ECG was recorded. Blood samples were collected for determination of the presence of anti-drug antibodies (ADAs) using an enzyme-linked immunosorbent or electrochemiluminescent assay [12].

### Statistical methods

Sample size calculations were based on an assumed change from baseline to week 13 in mean ACQ-6 score of −0.4 to −0.5 for placebo and −0.9 to −1.1 for the MEDI-528 groups, with a common standard deviation (SD) of 0.8 to 1.0 [15,16]. Sixty subjects in each treatment group would allow detection of a statistically significant difference between treatment groups with 58–99% power based on a 2-sided type I error of 0.05 and 0.10. Assuming a drop-out rate of 20%, it was planned to randomize 80 subjects into each treatment group.

The intent-to-treat (ITT) population was defined as all randomized subjects who received either placebo or MEDI-528. The safety population included all subjects who received either placebo or MEDI-528 and had safety data. Statistical analysis of clinical activity was based on a comparison of placebo with the individual MEDI-528 treatment groups for the ITT population.

Change from baseline (week 0) in mean ACQ-6 score at weeks 13 (primary endpoint) and 25 was analyzed using an analysis of covariance model. The model included the treatment group, atopic asthma status, geographic region, baseline eosinophil level, and mean ACQ score at baseline. Any missing ACQ-6 scores at weeks 13 and 25 were imputed using the last observation carried forward method. A sensitivity analysis was performed after excluding subjects with missing ACQ-6 scores at weeks 13 and 25. The proportion of subjects with an improvement in mean ACQ-6 score of ≥ 0.5 at weeks 13 and 25 was compared between placebo and MEDI-528 using Fisher’s exact test. Subgroup analyses were conducted on the primary efficacy outcome by atopic asthma status (atopic asthma, non-atopic asthma) and baseline use of ICS (medium dose, high dose). A subgroup analysis was conducted on the primary efficacy outcome by peripheral blood eosinophil count < 0.3 × 10^3^/μL or ≥ 0.3 × 10^3^/μL.

Weighted asthma exacerbation rate and the 95% confidence interval (CI) were calculated as the total number of exacerbations/total duration of person-year follow-up. The weighted asthma exacerbation rate at week 25 was analyzed using a pair-wise Poisson regression. Change from baseline in pre-bronchodilator FEV_1_ at week 25 was compared between placebo and MEDI-528 using a pair-wise t-test. The proportion of subjects with an improvement in overall AQLQ(S) score of ≥ 0.5 at weeks 12 and 25 was compared between placebo and MEDI-528 using Fisher’s exact test. Safety variables were analyzed descriptively based on the safety population.

## Results

### Subjects

**Figure 2:**
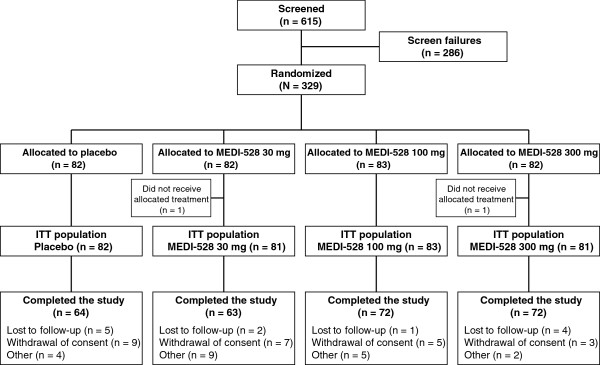
Subject disposition.

Of the 329 randomized subjects, 327 received either placebo or MEDI-528 and were included in the ITT and safety populations. Overall, 64/82 (78.0%) of the placebo-treated and 207/245 (84.5%) of the MEDI-528 treated subjects completed the study. The most common reason for discontinuation was withdrawal of consent (Figure 2).

Demographic characteristics were generally balanced between the groups (Table 1). The study population was predominantly female (69%) with a mean age of 43.0 years (range 18–65). Most subjects (84.4%) had atopic asthma and 50.3% had evidence of a peripheral blood eosinophilia (eosinophil count ≥ 0.3 × 10^3^/μL). Mean baseline ACQ-6 scores (2.8 for both placebo and combined MEDI-528 groups) indicated uncontrolled asthma, although 4 subjects (placebo, n = 1; 30 mg MEDI-528, n = 3) had a mean ACQ-6 score < 1.5 suggesting that their asthma was more controlled. Almost all subjects had experienced ≥ 1 exacerbation requiring oral steroids in the past year (placebo 80/82, 97.6%; MEDI-528 234/244, 95.9%). At baseline, an average of 2.06 exacerbations/subject required oral steroids during the previous year. Subjects in the placebo group appeared to have less severe asthma than those in the MEDI-528 groups, according to the proportion taking high-dose ICS (47.6% vs 52.2%) and rescue medication use (mean number of puffs per day 4.3 vs 5.1), although there were no statistically significant differences between placebo and any of the three MEDI-528 groups.

**Table 1:** Demographic and baseline asthma characteristics (ITT Population)

**Variable**		**MEDI-528**				***P *value**^**a**^
	**Placebo**	**30 mg**	**100 mg**	**300 mg**	**Combined**	
	**(n = 82)**	**(n = 81)**	**(n = 83)**	**(n = 81)**	**(n = 245)**	
Age, years, mean (SD)	43.6 (11.6)	41.8 (11.1)	45.1 (11.6)	41.5 (12.3)	42.8 (11.7)	0.16
Male, n (%)	29 (35.4)	26 (32.1)	21 (25.3)	26 (32.1)	73 (29.8)	0.56
Race, n (%)						0.62
White	42 (51.2)	34 (42.0)	33 (39.8)	40 (49.4)	107 (43.7)	
Asian	18 (22.0)	17 (21.0)	25 (30.1)	18 (22.2)	60 (24.5)	
American Indian or Alaskan Native	5 (6.1)	8 (9.9)	8 (9.6)	4 (4.9)	20 (8.2)	
Black or African American	2 (2.4)	3 (3.7)	4 (4.8)	5 (6.2)	12 (4.9)	
Mixed race	1 (1.2)	0 (0.0)	0 (0.0)	2 (2.5)	2 (0.8)	
Other	14 (17.1)	19 (23.5)	13 (15.7)	12 (14.8)	44 (18.0)	
BMI, kg/m^2^, mean (SD)	27.6 (4.3)	27.1 (4.4)	26.9 (4.3)	27.2 (4.4)	27.1 (4.3)	0.79
FEV_1_ (L)	2.21 (0.74)	2.22 (0.71)	2.05 (0.62)	2.27 (0.66)^b^	2.18 (0.67)^c^	0.19
FEV_1_, % predicted	70.7 (15.9)	71.8 (17.6)	70.6 (16.6)	72.2 (15.9)^b^	71.5 (16.7)^c^	0.90
FVC, % predicted	83.9 (14.6)	86.1 (16.7)	84.8 (14.3)	84.7 (14.8)	85.2 (15.2)	0.83
FEV_1_/FVC	68.0 (10.4)	67.6 (10.8)	67.0 (11.3)	69.4 (10.8)^b^	68.0 (11.0)^c^	0.57
FEV_1_ reversibility, %, mean (SD)	25.2 (13.8)^b^	23.9 (10.2)^d^	25.6 (15.3)^d^	21.8 (11.1)^e^	23.8 (12.5)^f^	0.25
Atopic asthma, n (%)	70 (85.4)	69 (85.2)	69 (83.1)	68 (84.0)	206 (84.1)	0.98
Number of exacerbations requiring oral steroids in past year, n (%)^g^						0.33
0	2 (2.4)	5 (6.2)	1 (1.2)	4 (4.9)	10 (4.1)	
1	45 (54.9)	47 (58.0)	42 (51.2)	39 (48.1)	128 (52.5)	
2	16 (19.5)	12 (14.8)	16 (19.5)	12 (14.8)	40 (16.4)	
3	5 (6.1)	5 (6.2)	10 (12.2)	15 (18.5)	30 (12.3)	
4+	14 (17.1)	12 (14.8)	13 (15.7)	11 (13.6)	36 (14.7)	
ICS use, n (%)						0.81
Medium dose	43 (52.4)	38 (46.9)	38 (45.8)	41 (50.6)	117 (47.8)	
High dose	39 (47.6)	43 (53.1)	45 (54.2)	40 (49.4)	128 (52.2)	
Rescue medication use, puffs per day, mean (SD)	4.3 (4.0)	4.9 (5.6)	4.5 (4.5)	5.9 (7.3)	5.1 (5.9)	0.25
Mean ACQ-6 score, mean (SD)	2.8 (0.7)	2.7 (0.9)	2.8 (0.7)	2.9 (0.9)	2.8 (0.8)	0.62
Overall AQLQ(S) score, mean (SD)	3.6 (0.8)^d^	3.8 (1.1)^h^	3.8 (0.9)^i^	3.8 (0.9)^h^	3.8 (0.9)^j^	0.60
Eosinophil count ≥ 0.3 × 10 ^3^/μL, n (%)	44 (53.7)	38 (47.5)^b^	43 (51.8)	39 (48.1)	120 (49.2)^c^	0.84
Eosinophil count (10^3^/μL)						0.47
Mean	0.40	0.34^b^	0.40	0.35	0.36^c^	
Median	0.31	0.28^b^	0.37	0.29	0.29^c^	
Basophil count (10^3^/μL)						0.05
Mean	0.03	0.03^b^	0.03	0.03	0.03^c^	
Median	0.03	0.03^b^	0.02	0.03	0.02^c^	

*Abbreviations*: *ACQ-6* Asthma Control Questionnaire, *AQLQ* Asthma Quality of Life Questionnaire, *BMI* Body mass index, *FEV*_*1*_ Forced expiratory volume in 1 second, *FVC* Forced vital capacity, *ICS* Inhaled corticosteroids, *ITT* Intent-to-treat.

^a^P value for differences among groups (placebo and MEDI-528 groups) by analysis of variance for continuous variables and the chi-square test for categorical variables.

^b^n = 80; ^c^n = 244; ^d^n=79; ^e^n=78; ^f^n=236; ^g^One value is missing in the MEDI-528 100 mg group; ^h^n = 76; ^i^n = 81; ^j^n = 233.

### Clinical activity

#### Mean ACQ-6 score

**Figure 3:**
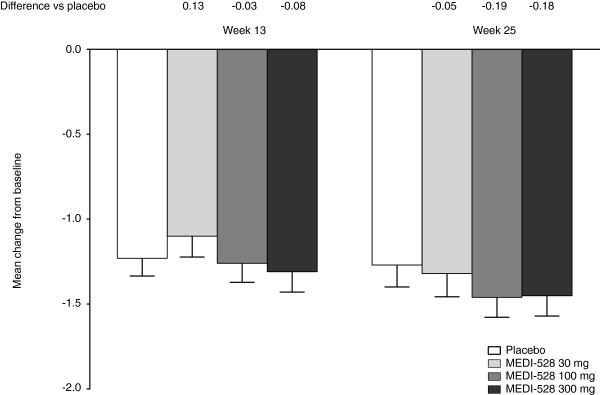
**Mean (SE) change from baseline in mean ACQ-6 score at weeks 13 and 25 (ITT population).** Abbreviations: ACQ-6, Asthma Control Questionnaire-6; ITT, Intent-to-treat; SE, Standard error.

The primary outcome measure analyzed using an analysis of covariance model, change from baseline to week 13 in mean ACQ scores, was not significantly different between the placebo and the individual MEDI-528 groups (p = 0.80). Improvements in mean ACQ-6 score occurred in all groups at weeks 13 and 25, with little difference between placebo and the individual MEDI-528 treatment groups (Figure 3). Clinically relevant improvements of ≥ 0.5 in mean ACQ-6 score occurred in similar proportions of placebo and combined MEDI-528 subjects at week 13 (64/82 [78.0%] vs 184/245 [75.1%], p = 0.66) and week 25 (59/82 [72.0%] vs 194/245 [79.2%], p = 0.22). In the individual MEDI-528 groups, a clinically relevant improvement was seen in the following proportions of subjects at week 13: 30 mg, 54/81 (66.7%); 100 mg, 66/83 (79.5%); and 300 mg, 64/81 (79.0%); and at week 25: 30 mg, 61/81 (75.3%); 100 mg, 67/83 (80.7%); and 300 mg, 66/81 (81.5%).

Differences between placebo and the combined MEDI-528 groups were not significantly different when analyzed by atopic or non-atopic asthma, baseline medium- or high-dose ICS use, or peripheral blood eosinophil count < 0.3 or ≥ 0.3 × 10^3^/μL (Table 2). In addition, there was no difference between the steroid-stable and steroid reduction phases with regard to the effect of ICS and systemic steroids (data not shown).

**Table 2:** ACQ score change from baseline to week 13 (ITT population)

**Variable**	**Placebo**		**Combined MEDI-528**		**P Value**^**b**^
	**n**	**Mean (SD)**	**n**	**Mean (SD)**	
All subjects^a^	82	−1.23 (0.95)	245	−1.22 (1.07)	0.86
Asthma type^a^					
Atopic	70	−1.28 (0.97)	206	−1.19 (1.04)	0.55
Non-atopic	12	−0.94 (0.82)	39	−1.42 (1.18)	0.41
ICS use^a^					
Medium dose	43	−1.21 (0.97)	117	−1.17 (1.02)	0.55
High dose	39	−1.26 (0.94)	128	−1.28 (1.11)	0.77
Eosinophil count^a^					
≥ 0.3 × 10^3^/μL	44	−1.39 (0.95)	120	−1.17 (1.01)	0.14
< 0.3 × 10^3^/μL	38	−1.04 (0.92)	124	−1.26 (1.09)	0.22
All subjects without imputation	67	−1.30 (0.94)	208	−1.28 (1.10)	0.75

*Abbreviations*: *ACQ* Asthma Control Questionnaire, *ICS* Inhaled corticosteroid.

^a^Missing mean ACQ score at Week 13 was imputed using the last observation carried forward method.

^b^P-values are calculated using an analysis of covariance with treatment and baseline value as covariates.

#### Asthma exacerbation rate

**Figure 4:**
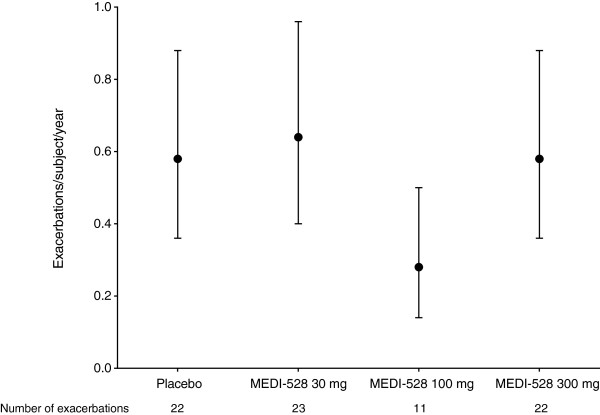
**Weighted rate of asthma exacerbation (95% CI) at week 25 (ITT population).** Abbreviation: ITT, Intent-to-treat.

At week 25, the weighted asthma exacerbation rate (exacerbations/subject/year, 95% CI) for the placebo group was 0.58 (0.36 − 0.88) compared with 0.49 (0.37 − 0.64) in the combined MEDI-528 groups (p = 0.52). Results were similar for the individual MEDI-528 dose groups (Figure 4); weighted asthma exacerbation rates (95% CI) at week 25 were 0.64 (0.40 − 0.96) (p = 0.76) for 30 mg, 0.28 (0.14 − 0.50) (p = 0.05) for 100 mg, and 0.58 (0.36 − 0.88) (p = 1.00) for 300 mg.

#### Pre-bronchodilator FEV_1_

**Figure 5:**
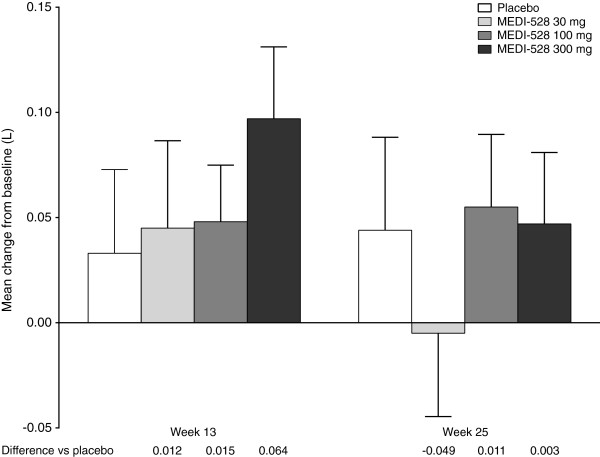
**Mean (SE) change from baseline in pre-bronchodilator FEV1 at week s 13 and 25 (ITT population).** Abbreviations: FEV_1_, Forced expiratory volume in 1 second; ITT, Intent-to-treat; SE, Standard error.

Mean increase from baseline in pre-bronchodilator FEV_1_ was similar between the placebo and MEDI-528 groups at weeks 13 and 25 (Figure 5). The FEV_1_ % predicted mean (SD) change from baseline at week 25 for placebo vs the combined MEDI-528 groups was 1.44% (11.08) vs 1.12% (9.81) (p = 0.82); mean (SD) change in FEV_1_ absolute values were 0.04 L (0.37) vs 0.03 L (0.30) (p = 0.82). In the individual MEDI-528 dose groups, mean (SD) change from baseline at week 25 was −0.01 L (0.32) (p = 0.41) for 30 mg, 0.06 L (0.30) (p = 0.85) for 100 mg, and 0.05 L (0.29) (p = 0.97) for 300 mg.

### Health-related quality of life

A clinically relevant improvement of ≥ 0.5 in overall AQLQ(S) score was seen in similar proportions of placebo and MEDI-528 subjects at week 12 (53/69, 76.8% vs 144/207, 69.6%; p = 0.21) and week 25 (50/62, 80.6% vs 136/182, 74.7%; p = 0.57).

### Safety

The proportion of subjects who reported at least one AE was similar between the placebo (68/82, 82.9%) and MEDI-528 (30 mg: 62/81, 76.5%; 100 mg: 68/83, 81.9%; 300 mg: 69/81, 85.2%) groups. The most frequent AEs in the combined MEDI-528 groups were asthma, upper respiratory tract infection, and headache (Table 3). Most AEs were mild or moderate in severity. One subject in the MEDI-528 100 mg group had a mild hypersensitivity reaction on the day of dosing that was considered possibly related to study medication and resolved the following day.

**Table 3:** **Adverse events that occurred in ≥ 2**% **of the combined MEDI-528 group (safety population)**

**Adverse event, n (%) of subjects**		**MEDI-528**			
	**Placebo**	**30 mg**	**100 mg**	**300 mg**	**Combined**
	**(n = 82)**	**(n = 81)**	**(n = 83)**	**(n = 81)**	**(n = 245)**
Asthma	25 (30.5)	30 (37.0)	21 (25.3)	31 (38.3)	82 (33.5)
Upper respiratory tract infection	12 (14.6)	6 (7.4)	16 (19.3)	20 (24.7)	42 (17.1)
Headache	8 (9.8)	13 (16.0)	5 (6.0)	6 (7.4)	24 (9.8)
Nasopharyngitis	12 (14.6)	11 (13.6)	5 (6.0)	7 (8.6)	23 (9.4)
Sinusitis	4 (4.9)	10 (12.3)	4 (4.8)	6 (7.4)	20 (8.2)
Influenza	6 (7.3)	10 (12.3)	5 (6.0)	2 (2.5)	17 (6.9)
Urinary tract infection	6 (7.3)	7 (8.6)	7 (8.4)	2 (2.5)	16 (6.5)
Pharyngitis	2 (2.4)	4 (4.9)	8 (9.6)	4 (4.9)	16 (6.5)
Bronchitis	8 (9.8)	4 (4.9)	2 (2.4)	5 (6.2)	11 (4.5)
Injection site erythema	2 (2.4)	1 (1.2)	4 (4.8)	5 (6.2)	10 (4.1)
Allergic rhinitis	7 (8.5)	5 (6.2)	1 (1.2)	3 (3.7)	9 (3.7)
Gastroenteritis	3 (3.7)	2 (2.5)	5 (6.0)	2 (2.5)	9 (3.7)
Diarrhea	2 (2.4)	3 (3.7)	3 (3.6)	3 (3.7)	9 (3.7)
Rhinitis	5 (6.1)	3 (3.7)	3 (3.6)	2 (2.5)	8 (3.3)
Back pain	2 (2.4)	3 (3.7)	3 (3.6)	2 (2.5)	8 (3.3)
Pneumonia	0 (0.0)	5 (6.2)	0 (0.0)	3 (3.7)	8 (3.3)
Dizziness	5 (6.1)	2 (2.5)	5 (6.0)	0 (0.0)	7 (2.9)
Injection site pain	5 (6.1)	1 (1.2)	4 (4.8)	2 (2.5)	7 (2.9)
Rash	3 (3.7)	2 (2.5)	1 (1.2)	4 (4.9)	7 (2.9)
Acute sinusitis	2 (2.4)	3 (3.7)	2 (2.4)	2 (2.5)	7 (2.9)
Hypertension	2 (2.4)	4 (4.9)	3 (3.6)	0 (0.0)	7 (2.9)
Pain in extremity	2 (2.4)	1 (1.2)	3 (3.6)	3 (3.7)	7 (2.9)
Cough	0 (0.0)	2 (2.5)	2 (2.4)	3 (3.7)	7 (2.9)
Oropharyngeal pain	0 (0.0)	2 (2.5)	3 (3.6)	2 (2.5)	7 (2.9)
Pyrexia	0 (0.0)	0 (0.0)	4 (4.8)	3 (3.7)	7 (2.9)
Migraine	1 (1.2)	4 (4.9)	1 (1.2)	1 (1.2)	6 (2.4)
Pruritus	1 (1.2)	1 (1.2)	2 (2.4)	3 (3.7)	6 (2.4)
Toothache	1 (1.2)	2 (2.5)	4 (4.8)	0 (0.0)	6 (2.4)
Nasal congestion	0 (0.0)	2 (2.5)	3 (3.6)	1 (1.2)	6 (2.4)
Pharyngotonsillitis	0 (0.0)	5 (6.2)	0 (0.0)	1 (1.2)	6 (2.4)
Bacterial upper respiratory tract infection	0 (0.0)	2 (2.5)	2 (2.4)	2 (2.5)	6 (2.4)
Asthenia	2 (2.4)	3 (3.7)	1 (1.2)	1 (1.2)	5 (2.0)
Malaise	1 (1.2)	0 (0.0)	3 (3.6)	2 (2.5)	5 (2.0)
Dyspepsia	0 (0.0)	3 (3.7)	1 (1.2)	1 (1.2)	5 (2.0)
Musculoskeletal pain	0 (0.0)	1 (1.2)	4 (4.8)	0 (0.0)	5 (2.0)
Bacterial pharyngitis	0 (0.0)	2 (2.5)	1 (1.2)	2 (2.5)	5 (2.0)

SAEs were reported by 4 (4.9%) and 15 (6.1%) subjects in the placebo and combined MEDI-528 groups, respectively (Table 4). Two SAEs (pneumonia and asthma) in one subject who received 30 mg MEDI-528 were considered to be possibly related to study medication. Six subjects discontinued the study due to AEs: one placebo (rash); two 30 mg MEDI-528 (ischemic stroke, headache); one 100 mg MEDI-528 (musculoskeletal pain and pain in extremity); and two 300 mg MEDI-528 (headache, bladder transitional cell carcinoma). Three of the AEs that resulted in discontinuation were considered likely to be related to study medication (100 mg, musculoskeletal pain and pain in extremity; 300 mg, headache).

**Table 4:** Serious adverse events by treatment group (safety population)

**Serious adverse event, n (%) of subjects**		**MEDI-528**			
	**Placebo**	**30 mg**	**100 mg**	**300 mg**	**Combined**
	**(n = 82)**	**(n = 81)**	**(n = 83)**	**(n = 81)**	**(n = 245)**
Asthma	0 (0.0)	6 (7.4)	1 (1.2)	2 (2.5)	9 (3.7)
Pneumonia	0 (0.0)	3 (3.7)	0 (0.0)	0 (0.0)	3 (1.2)
Food allergy	0 (0.0)	1 (1.2)	0 (0.0)	0 (0.0)	1 (0.4)
Bronchitis	0 (0.0)	1 (1.2)	0 (0.0)	0 (0.0)	1 (0.4)
Lobar pneumonia	0 (0.0)	0 (0.0)	1 (1.2)	0 (0.0)	1 (0.4)
Limb traumatic amputation	0 (0.0)	1 (1.2)	0 (0.0)	0 (0.0)	1 (0.4)
Thoracic vertebral fracture	0 (0.0)	0 (0.0)	1 (1.2)	0 (0.0)	1 (0.4)
Muscle spasms	0 (0.0)	0 (0.0)	1 (1.2)	0 (0.0)	1 (0.4)
Uterine leiomyoma	0 (0.0)	0 (0.0)	1 (1.2)	0 (0.0)	1 (0.4)
Complicated migraine	0 (0.0)	1 (1.2)	0 (0.0)	0 (0.0)	1 (0.4)
Ischemic stroke	0 (0.0)	1 (1.2)	0 (0.0)	0 (0.0)	1 (0.4)
Vaginal hemorrhage	0 (0.0)	0 (0.0)	1 (1.2)	0 (0.0)	1 (0.4)
Pancreatitis	1 (1.2)	0 (0.0)	0 (0.0)	0 (0.0)	0 (0.0)
Cholelithiasis	1 (1.2)	0 (0.0)	0 (0.0)	0 (0.0)	0 (0.0)
Hydrocholecystis	1 (1.2)	0 (0.0)	0 (0.0)	0 (0.0)	0 (0.0)
Appendicitis	1 (1.2)	0 (0.0)	0 (0.0)	0 (0.0)	0 (0.0)
Nephrolithiasis	1 (1.2)	0 (0.0)	0 (0.0)	0 (0.0)	0 (0.0)

No clinically relevant changes occurred in clinical laboratory values, vital signs, or ECGs.

ADAs occurred in 4/82 subjects (4.9%) in the placebo group and 22/245 subjects (9.0%) in the MEDI-528 groups (30 mg: 15/81, 18.5%; 100 mg: 4/83, 4.8%; 300 mg: 3/81, 3.7%). ADAs were detected prior to day 0 in 1 subject in the placebo group and 14 subjects in the MEDI-528 group.

## Discussion

This is the largest double-blind, placebo-controlled, dose-ranging study performed to date to determine whether an anti-IL-9 monoclonal antibody has any clinical benefits in subjects with poorly controlled, moderate-to-severe asthma. The results indicate that adding MEDI-528 to existing controller medications was not associated with any major safety concerns, but failed to achieve a clinically important effect on mean ACQ-6 scores at 13 weeks or on asthma exacerbation rates, lung function, or asthma-related quality of life at the pre-specified time points. However, this study was powered to detect a change in the primary study endpoint of mean ACQ-6 score from baseline to week 13, thus statistical results for the secondary endpoints must be viewed in this context.

Data from preclinical studies provide strong evidence that an IL-9 mast cell axis regulates airway inflammation, mucus production, airway hyperresponsiveness, and subepithelial fibrosis, with increased IL-9 expression in the airways in humans with asthma [5]. For example, both Th9 and Th17 are involved in asthma pathogenesis and produce IL-9 [17]. Kim et al. demonstrated in Balb/c mice that cellular infiltration related to chronic airway inflammation and remodeling can be reduced by an anti-IL-9 antibody. In addition, the number of Th9 cells, Th17 cells, mast cells, eosinophils, and neutrophils in the airway were reduced; the synthesis and secretion of cytokines was inhibited; and IgE synthesis in B cells was reduced [18]. These reported observations support our hypothesis that targeting IL-9 via an anti-IL-9 mAb would be an effective treatment for patients with poorly controlled asthma. Despite convincing *in vitro* and animal model data, there have been very few studies in humans examining IL-9 involvement in asthma.

In a previous small study in subjects with mild asthma, fewer subjects experienced ≥ 1 asthma exacerbation in the combined MEDI-528 group (1/27) compared with the placebo group (2/9; p = 0.15). The subject in the MEDI-528 group who experienced an exacerbation received the lowest dose (0.3 mg/kg), which indicated that it was not due to a dose effect. These results suggested a potential improvement in asthma exacerbation rates in subjects with mild asthma [11]. However, in the current study, no significant differences in the asthma exacerbation rate occurred between placebo and MEDI-528 in adults with uncontrolled moderate-to-severe persistent asthma. Likewise, the mean increase from baseline in pre-bronchodilator FEV_1_ was similar between the MEDI-528 and placebo groups, which is consistent with the earlier study where FEV_1_ was essentially unchanged and short-acting beta agonist use was comparable among groups [11]. Studies with other monoclonal antibodies, including omalizumab [19] and mepolizumab [20], also reported modest, if any, improvements in FEV_1_.

Although the study did not meet its primary endpoint, all groups, including placebo, demonstrated improvements in mean ACQ-6 score at 13 weeks. The placebo response has been well described in asthma studies and various explanations have been proposed. For example, Wise et al. reported that the placebo effect on asthma symptoms could be augmented by an optimistic message from the physician that enhanced the subject’s expectation of benefit from the drug [21]. Similarly, a large placebo response was observed in two pivotal studies of omalizumab and was explained by improved compliance with ICS treatment and/or intensive medical input [22].

It is becoming increasingly apparent that asthma is a heterogeneous disease, and that identification of potential subgroups or individual subject characteristics is likely to be key in delivering optimal response with therapeutics such as MEDI-528 that target specific immunological mechanisms. Subgroup analyses of the mean ACQ-6 score results provided no evidence that MEDI-528 may be effective in subjects with atopic asthma, peripheral blood eosinophilia, or in those taking moderate or high doses of ICS at entry to the study. Interpretation of these results should take into account the small numbers of subjects in some subgroups.

The heterogeneous nature of asthma makes it difficult for a targeted therapy with monoclonal antibodies such as MEDI-528 to show significant beneficial effects in a non-selected asthma population. Identification of biological markers may be of great help in determining more accurate asthma diagnosis and severity, predicting treatment response, or monitoring of disease control. Corren et al. showed that lebrikizumab increased FEV_1_ and reduced exacerbations in the high Th2 subgroup vs placebo [23]. Unfortunately, a pharmacodynamic marker for the IL-9 pathway has not been identified and IL-9 levels couldn’t be measured in blood or sputum in this study. Furthermore, subgroup analyses of the primary endpoint in pre-specified subpopulations, stratified by atopic status or medium- vs high-dose ICS at screening, and other pre-specified subpopulations based on FEV_1_, ACQ score, reversibility, and blood eosinophilia at randomization did not identify a subpopulation with improved efficacy over placebo.

Overall, the safety profile of MEDI-528 was similar to earlier clinical studies [11,12] and no new safety concerns were identified. ADAs were detected in the placebo and all three MEDI-528 treatment groups. None of these events were considered to be serious, but it is unclear why ADAs were detected in the placebo group or in some subjects prior to administration of MEDI-528. Our ADA assays may nonspecifically detect cross-reacting antigen or other epitopes; however, it is not uncommon for subjects to develop antibodies against a biologic agent [24].

## Conclusions

This study was designed to further evaluate the clinical effects of MEDI-528, an anti-IL-9 antibody, in light of the inconclusive results obtained in previous small studies. In this large study, we clearly demonstrated that adding MEDI-528 to existing controller medications was not associated with an improvement in ACQ-6 score, asthma exacerbation rate, FEV_1_, health-related quality of life, or any major safety concerns.

## Abbreviations

ACQ-6: Asthma control questionnaire-6; ADA: Anti-drug antibody; AE: Adverse event; AQLQ(S): Asthma quality of life questionnaire (Standardized version); CI: Confidence interval; ECG: Electrocardiogram; FEV1: Forced expiratory volume in 1 second; ICS: Inhaled corticosteroids; IL: Interleukin; ITT: Intent-to-treat; PEF: Peak expiratory flow; SAE: Serious adverse event; SD: Standard deviation; Th2: T helper cells.

## Competing interests

Chad Oh and Nestor Molfino were employed by MedImmune during the conduct of the study and owned stock in AstraZeneca. Micki Hultquist, Kimmie McLaurin, and Keunpyo Kim are employees of MedImmune and own stock in AstraZeneca. Richard Leigh has received research funding from MedImmune, payable to the University of Calgary, for conducting this and other clinical studies over the past 3 years.

## Authors’ contributions

CKO participated in design and coordination of the study. RL was a principal investigator for the study. KKM participated in the design of the study and interpretation of results. KK participated in the design of the study and performed the statistical analysis. MH participated in study design and interpretation of results. NAM conceived the study and participated in its design and coordination. All authors have been involved in drafting the manuscript or revising it critically for important intellectual content, read, and approved the final manuscript.
